# HIV and infant feeding in Malawi: public health simplicity in complex social and cultural contexts

**DOI:** 10.1186/1471-2458-12-700

**Published:** 2012-08-28

**Authors:** Jacqueline R Chinkonde, Marit Helene Hem, Johanne Sundby

**Affiliations:** 1 Institute of Health and Society, Department of Community Medicine, University of Oslo, Lilongwe, Norway; 2 Institute of Health and Society, Center for Medical Ethics, University of Oslo, Lilongwe, Norway

## Abstract

**Background:**

The question of when and how to best wean infants born to mothers with HIV requires complex answers. There are clinical guidelines on best approaches but limitations persist when applying them in diverse low-income settings. In such settings, infant-feeding practices are not only dependent on individual women’s choices but are also subject to social and cultural pressures. However, when developing infant-feeding policies little attention has been paid to these pressures, even though they may yield useful empirical knowledge on the various forces that shape the infant-feeding dilemmas confronting women with HIV. This study aimed to a) identify the infant-feeding challenges that women with HIV faced when they were advised to wean their children at an early age of six months and b) explore how the women adhered to their infant-feeding options while facing and managing these challenges.

**Methods:**

This study was conducted between February 2008 and April 2009 at two public health facilities where services to prevent mother-to-child transmission of HIV were implemented. Repeated in-depth interviews were conducted with 20 HIV-positive women. Two of the 20 women were also chosen for case studies which included home visits.

**Results:**

Several interdependent factors including the conflicting pressures of sexual morality and the demands of nurturing and motherhood, in conditions of abject poverty, impeded the participating women from following medical advice on infant feeding. If they adhered to the medical advice, the women would encounter difficulty maintaining their ascribed roles as respected wives, mothers and members of the society at large. The necessity of upholding their moral standing through continued breastfeeding, which signified HIV-negative status, put pressure on them to ignore the medical advice.

**Conclusions:**

The infant-feeding dilemmas for women with HIV are complex. The integration of public health efforts with context-specific socio-cultural understanding is essential. The recent 2010 WHO guidelines offer a possible way of resolving these challenges. They recommend breastfeeding for one year with an adaptation to two years for Malawi. Efforts in the PMTCT programmes to supplement existing support systems, e.g. through the mothers-to-mothers (M2M) programme or consultation with expert mothers may also help women overcome these challenges.

## Background

Breastfeeding is more or less universally practiced in most African countries like Malawi and is often prolonged beyond the age of 24 months [1-3]. Prolonged breastfeeding has been considered a good motherhood practice and vital to the survival of the newborn [4,5]. But for women with HIV, the fear of HIV transmission through breastfeeding is undermining this practice. Globally, a third of the children who become HIV-positive are infected through breastfeeding, and the remaining two-thirds during pregnancy and delivery [6,7]. However, HIV transmission through breastfeeding accounts for over 40% of all MTCT in sub-Saharan Africa [6-8]. High prevalence of HIV and the prevailing culture of breastfeeding are considered risk factors contributing to the high rates of MTCT in this region [9].

At the same time, due to poverty and a host of other related factors, non-breastfeeding can also threaten child survival. A number of new studies conducted in resource-poor settings like Malawi have raised concerns of heightened mortality and morbidity from diarrhoea and other infectious diseases associated with the avoidance or early cessation of breastfeeding as part of an HIV prevention strategy [3,10-15]. Poverty is rampant in Malawi with nearly 52% of the population living below the poverty line and 22% in extreme poverty. Between 70% and 85% of Malawian households encounter food shortages every year [16,17]. The scale of this problem has created a situation where young children must be fed whatever foods are available, making it impossible to buy healthful milk substitutes. Infant feeding is a challenge regardless of HIV status. An estimated 13% of Malawian under-five children are currently underweight, 5% are wasted and 46% are stunted [3]. To minimize these challenges, breastfeeding is still promoted as the main source of child nutrition with the potential to prevent about 13-15% of all under-five deaths [4,11,18]. Thus, the overarching concern for women with HIV lies in the need to minimize the risks of MTCT without undermining the benefits of breast milk.

In responding to these challenges, the United Nations member organizations (UNAIDS, WHO, UNFPA and UNICEF) have offered guidance in the development, adoption and adaptation of HIV and infant-feeding clinical guidelines. Emphasis has been given to new scientific information that has become available. In resource-rich settings, wide use of highly active antiretroviral therapy (HAART) during labour and child birth, caesarean delivery and formula feeding have lowered the levels of MTCT to under 2% [19]. Finding the optimal infant-feeding strategy for women with HIV in resource-poor settings like Malawi where there is often limited access to HAART remains a complex issue [8]. In such settings, clinical guidelines have fluctuated between exclusive formula feeding, exclusive breastfeeding in the first six months of life, and, most recently, providing antiretroviral (ARV) drugs to either the mother or the child until one week after exposure to breast milk has ended [7,8,20-23]. In addition to the aforementioned challenges related to replacement feeding, exclusive breastfeeding has also proved difficult to adhere to even though most mothers breastfeed for nearly two years [24].

Most PMTCT programmes in Africa, including Malawi, have also provided infant-feeding counseling on a one-on-one basis to the baby’s birth mother. This individual approach has given the women the chance to freely decide the most appropriate alternative on their own [25]. However, it has paid little attention to other important influences beside the mother/child relationship, which are beyond the mothers’ control, but which may be essential for adherence to the chosen options [26-28]. Studies conducted in a number of African countries like Tanzania, Malawi and Uganda, for instance, have highlighted the important roles played by male partners, grandmothers and/or mothers-in-law in influencing infant-feeding decisions [16,26,29]. Thus, in addition to the threat that HIV poses to child survival, it also affects the cultural conditioning and social relationships of HIV-infected women. In this case, ensuring safe infant feeding practices means finding a strategy that takes into account discrepancies between the counselling provided to the women and conduct that is socially and culturally preferable. Although it is expected that individual counselling should provide for this that has not been the case. Reports have shown that health workers have mostly encouraged the women to stop breastfeeding at six months without necessarily helping them weigh the risks and benefits of their chosen options [30,31].

We have conducted several studies on breastfeeding and infant feeding in the context of HIV/AIDS [30,31]. In our research we found that not much was known in Malawi about the socio-cultural and economic dimensions of infant feeding. In particular, the moral issues underlying breastfeeding needed to be explored. Limited information also existed on the tensions and interactions between the bio-medical recommendations and the socio-cultural and economic dimensions of infant feeding. The aims of this study were to: a) identify the infant-feeding challenges that women with HIV faced when they were advised to wean their children at an early age of six months and; b) explore how the women adhered to their infant-feeding options while facing and managing these challenges. This paper seeks to examine some of the complexities underlying the formulation of practices in a context where the public health message is believed to be pragmatic and straightforward: “you either do or you don’t”. It describes some of the socio-economic limitations on poor women’s decision-making power and demonstrates how interrelatedness and cultural norms clash with the straightforward public-health advice.

## Methods

### Setting

This was a qualitative study that followed each mother-child pair over a period of 14 months, yet the selected period was most relevant for decision making on infant nutrition. The study was conducted between February 2008 and April 2009. Field work was carried out by the first author and a research assistant who was a nurse with a social science background. The sample was recruited from a large prospective study of HIV-positive women enrolled in a prevention of mother-to-child transmission (PMTCT) programme run by the University of North Carolina (UNC) project in Lilongwe, Malawi. In collaboration with the Malawi Ministry of Health (MoH), UNC began providing PMTCT services in 2002 at four public health facilities in Lilongwe before the official launch of the programme in 2003 [30]. Over the years, the project has also provided technical assistance to 41 other public facilities in the district. This programme has been a pioneer programme for providing PMTCT interventions in Malawi.

The study was carried out at two of the first four PMTCT facilities: the rural Area 25 health center and the semi-urban Bwaila hospital. Bwaila has a catchment population of just over 500,000 people, provides antenatal care services to nearly 60,000 pregnant women and conducts up to 10,000 deliveries a year. Due to the effects of migration, the population is a multi-ethnic mix of socio-culturally heterogeneous groups. Area 25 mainly serves the indigenous small-scale farmers of Chewa origin, the main ethnic group in Malawi. However, it is not unusual to also find other tribes because of relocations from town in pursuit of more land. Area 25 serves an estimated 100,000 people, provides antenatal care to nearly 5,500 newly pregnant women annually and conducts up to 3,000 deliveries each year. In general, Area 25 and Bwaila hospital both have high ANC service participation although the proportion of facility births is relatively low. As with most parts of Malawi, breastfeeding rates are high with nearly 99% of children having been breastfed at some point [3]. Mixed feeding patterns are also common with the early introduction of other foods and fluids besides breastfeeding [3,16]. In both facilities, as with the rest of Malawi, husbands play important roles in decisions related to reproduction and family health, which puts women in subordinate positions [32,33].

### Sample, data collection procedure and data analysis

The core participants were lactating HIV-positive women who had children that were close to six months old and who had plans to wean these children at that age. They were initially contacted to join the study by programme staff during their follow-up to the programme. Of the 22 women approached, 20 agreed to participate. Repeated in-depth interviews were held using a semi-structured interview guide. The interviews were repeated because there was a need to explore the infant-feeding challenges at particular points in time such as at transition from breastfeeding to the introduction of other foods and during the first months of replacement feeding.

The questions mainly focused on the mother’s knowledge and beliefs about HIV and infant feeding, the type of infant-feeding counseling they had received, the women’s planned infant-feeding practices at age six months, problems foreseen or encountered with their chosen option and the ways in which they coped with those challenges.

As the interview sessions progressed, two of the 20 participating women were also chosen for case studies. This selection was made by both researchers during a third encounter with each woman when the patterns of what was happening in her life had started to emerge. Permission for home visits was also obtained at this time. Home visits were initiated because the home environment was more confidential and not as hectic as the clinic. The first case was chosen in order to learn more about poverty and the ways in which it manifested itself in the lives and feeding practices of the women. The second case provided insight into family dynamics, particularly gender inequality and the ways in which it influences the feeding practices of women with HIV. To get a balanced account on these issues, the views of the partner in the second case were also sought.

A total of 73 interviews were conducted with the women. Three of the women were interviewed only once, two of them three times and 15 of them four or five times. As mentioned before, there was an additional interview with one man. The first author’s years of experience as a service provider in this programme helped her communicate with the women and gain a better understanding of their everyday challenges from the “emic” or “experience near” perspective as opposed to the “etic” or “experience-distant” perspective [34,35].

All interviews were conducted in Chichewa Malawi’s local language, and were tape-recorded in circumstances where the informant provided consent. Each session lasted 40 to 60 minutes. The taped information was transcribed verbatim and translated from Chichewa into English on the same day of conducting the interviews. Each interview took 3–4 hours to transcribe. Using content analysis [35,36], local research staff read through the scripts independently to identify emerging themes from the text and/or to identify issues requiring clarification in subsequent interviews. Sections of text were then marked and cross-linked with similar text from the other interviews. The researchers then jointly identified recurrent themes related to the original research questions and categorized them accordingly. The categorized themes were then coded under each research question, entered into an electronic file and analyzed. The final analysis was summarized and presented in an analytical document that the research team and the co-authors commented on. The whole process involved working back and forth through the data material, being systematic and intuitive at the same time, and letting ideas from different sources guide the analysis as recommended by Kvale and Brinkman [35].

This study was conducted in accordance with the Helsinki Declaration. Ethical approval was granted by the Malawi National Health Sciences Research Committee (Protocol #443) and the Regional Committee for Medical and Health Research Ethics in South-Eastern Norway (Project number REK 2.2007.2374). Ethical issues involved in this study were concerned with performing home visits and conducting individual interviews with women on topics of a sensitive nature. Among the fundamental ethical research issues raised were those involving privacy and informed consent. Voluntary participation and the freedom to withdraw at any time as well as the assurance of anonymity and confidentiality were emphasized throughout the study [34,35].

### Results

The analysis identified several complex, interdependent factors that impeded the women from following the advice they were given on HIV and infant feeding. These are presented under the following themes: public policy confusion, HIV and its links with sexual morality and stigma, poverty and HIV-related feeding practices and sex and abstinence during the breastfeeding period. Coping mechanisms used by the women to overcome the challenges were also identified. These themes will be discussed in greater detail after a description of the respondents.

### Socio-demographic characteristics of the women

All of the women were married and housewives. Most of the marriages had been unstable, characterized by separation and re-union, divorce and frequent involvement of partners in multiple concurrent sexual relationships. Three women were in polygamous unions. Nearly all reported that they had become mothers within a year or two of their wedding. Table 1 below presents selected demographic characteristics of the participating women.

**Table 1:** Socio-demographic characteristics of the study participants

**Characteristic**	**Semi-urban (N = 10)**	**Rural (N = 10)**
Average age (range)	32.2 (20–42)	28.5 (21–36)
Average age at marriage (range)	18.3 (13–25)	20.2 (17–31)
Average years of schooling (range)	6.4 (1–9)	6.3 (3–9)
Average parity (range)	4 (2–8)	3.4 (1–6)

### Key challenges

#### Public policy confusion

All women in this study had received advice on HIV and infant feeding promoting exclusive breastfeeding in the first six months of the child’s life. However, there was confusion about how to feed the children after six months. All women had been advised at the PMTCT programme to wean their children at six months, but eight of the women had also attended the paediatric HIV clinic where they were advised to continue breastfeeding until their children were 12 months. The women’s concerns were expressed in statements such as: *“I am now confused because I have been told to wean at six months yet at the same time they* (health workers) *are also telling me to continue breastfeeding until my child is one year old”. “The health workers seem not to know what they are doing, they are giving us conflicting messages on infant feeding”. “I am worried that I may not be able to protect my child from acquiring HIV. I have received contradictory information on infant feeding”.*

The sources of these conflicting messages were identified as two different organizations that independently collaborated with the District Health Office (DHO). Lack of adequate cooperation was especially evident at Area 25 where health workers were noted using different versions of the WHO infant-feeding guidelines, one based on the 2001 and the other on the 2006 recommendations.

The women also reported insufficient support at the community level, which they felt was critical for sustaining the advice on infant feeding: *“We received this advice during our initial antenatal visits and again once or twice after delivery*. *There is nothing happening to support us in our communities”.* The confusion that arose from the conflicting advice and the limited support they were offered may explain why the women weaned at arbitrary time points so that there does not appear to be any logic as to why those dates were selected. Some weaned their children as early as 5 months whereas others mixed breast milk and other foods after the child turned six months.

#### HIV and its links with sexual morality and stigma

The study findings revealed breastfeeding as the only culturally acceptable method of infant feeding and the only way to fulfill the ideals of being a good mother. From the discussions with the women, it was obvious that any deviation from this breastfeeding norm in this community signaled an HIV-positive status, which in turn cast doubt on the woman’s sexual morality. When the women stopped breastfeeding at 6 months for example, it was assumed that they were doing so because they were HIV positive. As a result, they were blamed for that and were treated like loose women even though it may have been their husbands who brought the HIV into the marriage. As a result, all the 20 interviewed women said they had been berated when they chose to stop breastfeeding their children at the age of six months as illustrated by this quote: *“My friends insisted to know why I stopped breastfeeding my daughter at an early age of six months*. *Some said they had heard on the radio that anyone who is HIV positive should stop breastfeeding at six months. Others mocked me for having gained weight which they attributed to responding well to antiretroviral drugs. They even volunteered to accompany me on my next visit to the hospital to learn about my diagnosis.”*

Perhaps the more significant reactions were those of the participating women’s partners’ when their wives disclosed their HIV-positive status and attempted to stop breastfeeding their children at six months. Mrs. R recounted her husband’s reaction after she weaned her son at the age of six months, *‘He squeezed my breasts and forced me to breastfeed. When I resisted, he called me a witch for wishing to kill his child. He warned me that if I wished to stop breastfeeding I should do that at my mother’s place and not in his house’.* She was forced out of her husband’s house on two occasions, first when her son was six months old and again at 9 months. In this case, following the infant-feeding recommendations threatened her status as a morally acceptable mother and sex partner.

#### Poverty and HIV-related feeding practices

Whereas most participating women were determined to wean to avoid transmitting the virus to their children, their poverty and food insecurity played an extremely important role in determining how and when this could be achieved. Lack of adequate food was evident among the recently weaned children whose growth monitoring records showed significant faltering growth. The weight of Mrs. E’s son, for instance, dropped from 7.4 kg after she had weaned him at six months to about 7.1 kg at 10 months. The experiences of women whose children had been weaned at six months illustrated how difficult it was to wean at this early age. Some children were observed crying uncontrollably due to hunger, and others even snatched away other children’s snacks.

Due to the lack of adequate weaning foods, many women who otherwise wished to follow the recommended medical advice chose instead to delay weaning until they felt that their children could survive without breast milk. One woman, Mrs. A, whose child was 11 months old, described her assessment of the proper timing for weaning her son as follows, “I *plan to wean my child in March because that is when I will have enough food from my harvest to feed him”.*

It was noted during the home visits and discussions with the women that the food situation was worse for the semi-urban dwellers than it was for the rural counterparts. In this situation, the improvement was partially due to access to land for cultivation. The 20 women interviewed almost exclusively relied on their partners for financial support, but the partners were also poor. Most of the partners in the study earned their meagre income from informal employment and/or small-scale businesses. Mrs. R’s husband expressed concern about his economic situation: “*When my son attained the age of six months, I had just lost my job. I remained unemployed for almost a year. My monthly salary of 5000 Malawi kwacha (US$ 38) on the new job was not even enough to cover the transportation costs to get free food supplements at the prevention programme.* He had therefore forced his wife to continue breastfeeding due to their financial constraints.

The poverty was even worse for participants who did not disclose their HIV-positive status to partners. Without their partners’ support, they had exceptional difficulty allocating resources for replacement food or participating in the follow-up care. The major issue was that they had no convincing reason for their actions. Thus, few women with scarce resources were able to wean their children at the age of six months or a few months thereafter. Most were forced to wait until their children had grown older, often close to or beyond the age of 12 months. Mixed feeding was also common, with breastfeeding a part of this option for some.

#### Sex and abstinence during the breastfeeding period

The participating women also identified several parties that expected them to behave in a particular manner in caring for themselves, their children and their marriages; such as the medical community, their husbands and the society at large. At times, the perspectives of these groups were in conflict with each other, which placed the women in a precarious balancing act. The women for instance described being required by tradition to observe postpartum sexual abstinence during the breastfeeding period to avoid contaminating the breast milk with semen. It was feared that the semen would trigger diarrhoea and faltering growth in a suckling child. At the same time, they also reported being coerced by their partners to resume sex early. For fear of negative repercussions such as marital dissolution, nearly all of the study participants reported to have resumed sexual activity by the time the child was six months old. This was regarded with displeasure by the older women in the community. The following quote from a participant illustrates the older women’s reactions, *“The generation of mothers today is not heeding advice. They have ignored tradition. They are resuming sex even when their children are small and still breastfeeding. If one tries to reason with them, they question your instructions”.* They attributed the children’s increasing ill-health, such as weight loss, to the ‘toxic’ effects of ‘contaminated’ breast milk. HIV fits into the problem of early resumption of sex because HIV-positive women who try to wean at six months can argue that their husbands’ desire for sex was the reason why they weaned.

### Strategies used by women to overcome the challenges

Most mothers made attempts to govern the above challenges, attempting to protect their children’s health while also trying to evade social condemnation and stigmatization.

#### Protecting children’s health while evading the social condemnation

One of these evasive yet protective efforts is reflected in the case of Mrs. R. Confronted with the dilemma of passing on the virus to her child if she did not wean him, yet jeopardizing her moral status if she did, she said: *“Whenever I am in the company of friends and my child* (then 10 months old) *starts crying, I let him suckle the side of the breast to mimic breastfeeding. I then excuse myself to give him other foods in private”.* Others, like Mrs. J, had small-scale businesses that kept them away from home and friends while their children were left in the care of older siblings. By evening when they returned home, none of their friends would be around to observe their feeding practices. Still others carried their children to businesses, where they had the freedom to feed them on anything without their friends knowing.

#### Learning from experience

Others never bothered about the consequences that would result from following the recommended advice. All they cared about was ensuring that their children would be protected from becoming infected. It was noted that those who chose this path were previous programme participants who had subsequent births. Having witnessed the difficulties that their friends went through in caring for children who had become infected through breastfeeding, they felt that this superseded all other threats as Mrs. F described: “*I and my friend were first tested for HIV in 2004 when we both tested positive. After the health workers had told us that HIV can also be found in breast milk, we thought they were just cheating us, so we never took their advice seriously and practiced mixed feeding. I regret that now because my friend’s child is HIV positive and is taking ARVs. Although mine did not acquire the infection, I sympathize with my friend because her child is always ill. I will never repeat taking the advice from hospital lightly”.*

Mrs. F weaned her son at the age of exactly six months. By nine months he weighed 9 kg. Mrs. F acknowledged that she felt free to disclose her infant-feeding practices to anyone who cared to question them. She also acknowledged the support provided by her husband (also HIV positive), which encouraged her to overcome most of the challenges she encountered.

#### Other measures

Others, like Mrs. A, used force to silence those who had persistently gossiped about her being HIV positive. Still, it was surprising to note that some of the women, especially those who had developed physical illness, received material support from friends who had formerly gossiped about their HIV-positive status. Mrs. S, for instance, developed swollen legs which disabled her from acquiring free formula at the clinic and from participating in subsequent care. She had also recently been divorced and was left to care for two children, 26 and 11 months old, who weighed 5.8 kg and 7 kg respectively. Mrs. S was rescued by her social network, which unfortunately could only provide this assistance for a short time before they too became increasingly susceptible to poverty. Nevertheless, they advised her to continue breastfeeding the last born because they attributed the poor health of her next-to-the-last child to early weaning.

In summary, the findings above have provided insight into various interrelated factors contributing to the infant-feeding challenges affecting women with HIV. These are discussed in greater detail after the section on limitations.

### Limitations

The findings in this study were generated from two public health facilities, both of which were located within one city in central Malawi. As such, they apply to that context. However, the results of qualitative studies may often be generalized if the concepts found in one setting make sense in other similar settings [34]. Although the views of related men and other significant women were only explored to a limited extent in this study; their views were surveyed in the findings of a formative study that preceded this main study, so in that sense they informed the development of the interview guides.

## Discussion

Based on the findings of our interviews, we observed that the functioning PMTCT programmes promoted exclusive breastfeeding during the first six months of a child’s life. At the time of this study, rapid cessation of breastfeeding after 6 months was the internally recommended strategy. The changes in the guidelines occurred so rapidly that the system could not follow up quickly enough. Thus there were conflicting messages about feeding recommendations. The new 2010 recommendations, to breastfeed up to one year, will help alleviate some problems identified by HIV-positive women in Malawi.

In this study, several parties, including but not limited to the medical community, the husband and society-at-large expected the nursing mother to behave in a particular manner with respect to breastfeeding. Unfortunately, the advice given by health care providers occasionally conflicted with community views, placing the women in a no-win situation. While these challenges existed because of public policy confusion and traditional beliefs, issues like stigmatization and poverty/structural violence also played a role. In this paper, we chose to discuss the issue in light of different concepts of risk, and the balance between different “risks”.

It is noteworthy that most of the women attempted to follow the medical advice even when that advice was confusing and sometimes conflicting. As previous studies have suggested, a possible explanation for their persistence may relate to the authority that biomedicine has attained within the PMTCT programme and the corresponding focus on biomedical treatments, which tend to override women’s other needs [37]. Yet for the women, there are again many other forms of “risk”, some of which are not statistically measurable. The risks of social exclusion, stigmatization and damaged marital relationships are certainly real for them. These risks are perceived as “either-or” types of absolutes. These competing social risks based on local beliefs about motherhood and the accompanying socio-economic constraints give rise to infant-feeding dilemmas for the individual women.

In Malawi, breastfeeding is a behavior at the very heart of motherhood, which is regarded by many as the only acceptable way to feed a child. It is also noted that the women’s social networks including their partners and/or elderly women have played a significant role in influencing decisions related to infant feeding. Evidence from a number of African countries, including Tanzania, Uganda and Malawi, suggest close links between breastfeeding and sexuality, which have rendered the practice subject to control, power and authority [8,26,28,29,37].

As our findings clearly show, however, the women did not always make choices dictated by their social networks. Many mothers interviewed were more responsive to concerns associated with transmitting HIV to their children. They were responsive even though the bio-medical information conflicted with pre-existing cultural norms and was often provided by facilities in ways that were confusing and at times conflicting. Without adequate support at the community level, the women faced the challenge of having to resist the social pressures to breastfeed. This is evidenced by some reversion to breastfeeding even after the mothers had weaned their children. Similarly in Tanzania, Uganda and South Africa, social pressures to breastfeed have been important factors counteracting formula feeding [8,26,28,29].

It is even more notable that the women’s decisions as to whether to breastfeed were made in a context where HIV is also highly stigmatized and where non-breastfeeding might arouse suspicion in the community of an HIV- positive status [31,38]. Early weaning in this case risked involuntary disclosure of that status, which could stigmatize the woman in her community. Due to the limited attention paid to the concerns of the participants at the clinics [30,31], health workers were much too far removed from the specific circumstances affecting the women to be able to comprehend those circumstances. Within this setting, for example, breastfeeding after children had reached the age of six months implied a socio-economic status where the family was too poor to afford replacement food. Because the issue of malnutrition has also been highly prevalent and been given as much priority as HIV prevention, breastfeeding is still regarded as the most viable option for infant survival in spite of the MTCT risks [4,25,28]. The effect of the conflict between the medical advice and the local understanding of infant feeding has been to increase women’s concerns about protecting the well-being of their children, their social relationships and the existence of their marriages. It is possible that these conflicting views have mutually re-enforced each other in perpetuating the infant-feeding dilemmas for women with HIV.

## Conclusions

Efforts to minimize postnatal HIV transmission have been based on a biomedical interpretation of MTCT risks through breastfeeding and the recommended actions included weaning children at an early age of six months. However, this study has identified a number of challenges associated with the conflicts between the medical recommendations and local practices, where the latter have impeded women with HIV from adopting medical advice. The merging of sexual morality and the morality of nurturing and motherhood in conditions of abject poverty has subjected many of these women to enormous dilemmas. While attempting to adhere to the medical advice, the interviewed women struggled to adequately nurture their children, protect their marriages and maintain their social status. Non-adherence to advice has been perceived as a necessity to maintain their positions as wives and mothers and to fit into their broader social networks. Thus, the importance of upholding their moral standing through continued breastfeeding, which signals HIV-negative status, has conflicted with the medical advice.

There is a need for interdisciplinary approaches that integrate clinical guidelines with contextualized socio-cultural understandings in ways that are meaningful and inclusive and that bring benefits for all involved. The recent 2010 WHO guidelines on HIV and infant feeding, for instance, have recommended a breastfeeding period of one year, but for Malawi they have recommended lengthening that period to 2 years. The implication of the revised guidelines is that as long as one is on “Option B plus”, which is lifelong Highly Active Antiretroviral therapy (HAART) now in current use; breastfeeding still remains the method of choice. Early and rapid cessation is no longer recommended [18,39]. The Option B plus strategy is currently being rolled-out across most health facilities in Malawi. Operationally, some of the threats which have also been highlighted in the literature and in the Malawi national experience include suboptimal uptake of HAART by this relatively healthy population and poor follow-up [30,31].

The findings of other studies that the effectiveness of PMTCT programmes and maternal and child health services in general have been contingent upon male involvement and on adequately addressing the problem of food insecurity [4,32,40] are in agreement with the findings from our interviews. One pilot initiative in Malawi has also demonstrated the effectiveness of involving expert patients in PMTCT programmes in order to improve treatment uptake and trace clients on ART [41]. Likewise, the Mothers-to-Mothers programme has shown promise in improving the health outcomes of PMTCT clients. Although these programmes have not offered direct support in addressing infant-feeding challenges per se, lessons can be drawn that would help improve the coping strategies utilized by women such as those interviewed for the current study.

## Competing interests

The authors declare that they have no competing interests.

## Authors’ contributions

JRC conceived and designed this study, collected and analysed the data, and drafted the manuscript. JS and MHH contributed to the drafting and critical revision of the manuscript. All of the authors read and approved the final manuscript.

## Pre-publication history

The pre-publication history for this paper can be accessed here:

http://www.biomedcentral.com/1471-2458/12/700/prepub
